# Sterile insect technique and F1 sterility in the European grapevine moth, *Lobesia botrana*

**DOI:** 10.1093/jis/14.1.8

**Published:** 2014-01-01

**Authors:** George Saour

**Affiliations:** Department of Biotechnology, Atomic Energy Commission of Syria, P. O. Box 6091, Damascus, Syria

**Keywords:** gamma radiation, inherited sterility, pest management, radiation biology

## Abstract

Newly emerged adults of the European grapevine moth, *Lobesia botrana* (Denis and Schiffermuller) (Lepidoptera: Tortricidae), were irradiated with various doses of gamma radiation and crossed to unirradiated counterparts of the opposite sex. Fecundity was decreased when unirradiated females were mated with either 300-or 350-Gy-irradiated males. Adult males that were irradiated with 400 Gy and mated with unirradiated females retained a residual fertility of 2.7%. The radiation dose at which irradiated females were found to be 100% sterile when mated with unirradiated males was 150 Gy. The inherited effects in the F1 progeny of irradiated male parents were examined at 100, 150, and 200 Gy. Fecundity and fertility of the F1 progeny of males irradiated with 150 Gy and inbred or crossed with irradiated and unirradiated moths were also recorded. A significant reduction in fertility was observed when F1 males mated with either F1 or unirradiated females. According to sterility index, F1 females who mated with F1 males had greater sterility than when F1 females were crossed to 150-Gy-irradiated males. Based upon the results of this study, 150 Gy of gamma radiation would be the optimal dose to use in a sterile insect technique and F1 sterility program against *L* . *botrana* .

## Introduction

The European grapevine moth, *Lobesia botrana* (Denis and Schiffermuller) (Lepidoptera: Tortricidae), is a polyphagous insect that develops on more than 200 plant species from various families. It is one of the most serious pests in Mediterranean and southern European area vineyards ( Ifoulis and Savopoulou-Soultani 2004 ). Recently, *L* . *botrana* was inadvertently introduced to Japan and has been reported from Chile, Argentina, and the Napa Valley in California (USA) ( Varela et al. 2010 ). *Lobesia botrana* adults appear in vineyards in early spring. First-generation larvae feed on inflorescences, whereas 2 ^nd^ and 4 ^th^ generations damage the green, ripening, and ripe berries.

Several control tactics have been evaluated against *L* . *botrana* , including the use of insecticides, biological control agents, and mating disruption methods ( Ifoulis and Savopoulou-Soultani 2004 ; Irigaray et al. 2005 ; Akyol and Aslan 2010 ). It is well documented that the widespread use of insecticides is highly dangerous in sensitive ecosystems ( Yadav 2010 ). However, in most vineyards *L* . *botrana* is still controlled by using broad-spectrum chemical insecticides ( Irigaray et al. 2010 ; Ioriatti et al. 2011 ).

Research on the radiobiology of insects demonstrated that Lepidoptera and Homoptera are radiation-resistant orders and require a radiation dose much higher than that of other insect orders classed as radiation-sensitive (Barkri et al. 2005). A major difference between these two groups of insect orders is that the former group has a diffuse centromere (holokinetic) and the latter has a localized centromere (monokinetic) ( Murakami and Imai 1974 ). It is believed that the centromere difference could play a major role in radiation sensitivity ( Carpenter et al. 2005 ). However, it was suggested that possible molecular mechanisms responsible for the high radio-resistance in Lepidoptera might include an inducible cell recovery system and a DNA repair probe ( LaChance and Graham 1984 ). Another genetic phenomenon found in Lepidoptera is that females are more sensitive to radiation than are males of the same species, and inherited sterility in irradiated lepidopteran males is caused mainly by induced translocations that lead to the production of genetically unbalanced gametes in F1 individuals, resulting in their sterility ( Tothova and Marec 2001 ).

The use of autocidal control methods (i.e., sterile insect technique (SIT) and the phenomenon of inherited sterility or F1 sterility in Lepidoptera) represents an environmentally and medically benign option that offers great potential for managing *L* . *botrana* . The advantages of F1 sterility over the completely sterile insect in Lepidoptera pest control were largely discussed by several authors (lower doses of radiation used to induce F1 sterility increased the quality and competitiveness of the released moths) ( Makee and Saour 2004 ; Carpenter et al. 2005 ; Tate et al. 2007 ; Soopaya et al. 2011; Jang et al. 2012 ). Moreover, inherited sterility has been demonstrated in a number of economically important Lepidoptera. Sterile moths are currently being applied as part of operation area-wide integrated pest management programs for the following three lepidopteran pests: the codling moth, *Cydia pomonella* , in western Canada, the false codling moth, *Thaumatotibia* (= *Cryptophlebia* ) *leucotreta* , in South Africa, and the cactus moth, *Cactoblastis cactorum* , in the USA and Mexico ( Hight et al. 2005 ; Bloem et al. 2007 ; Carpenter et al. 2007 ).

There are no published data concerning the effects of gamma radiation or the use of SIT against *L* . *botrana* . Therefore, the purpose of this study was to examine the effect of various doses of gamma radiation on the fecundity and fertility of *L* . *botrana* when insects were inbred or crossed with unirradiated mates. Moreover, the minimum dose at which females were 100% sterile when mated with fertile males was determined. Based on the results of the first set of experiments on the parental generation, three doses were chosen for documentation of inherited sterility effects in this species. Mortality during development, sex ratio distortion, and fecundity and fertility of the F1 generation produced from irradiated males and unirradiated females were also determined. The results obtained are discussed in the context of using SIT/F1 sterility as a speciesspecific pest control tactic that could be used to eradicate or prevent further geographic range expansion of *L* . *botrana* .

## Materials and Methods

### Insects

Insects used in the experiments were obtained from a laboratory stock culture, which was renewed each year with field collected *L* . *botrana* larvae from infested grapevine. The larvae were reared on a semi-artificial diet as described in Thiery and Moreau (2005) at 25 ± 1º C, 60 ± 10% RH, with a photoperiod of 15:8 L:D and 1 hr of dusk. Male and female adults (100 pairs) were placed in a large cage (30 x 60 x 30 cm), furnished with bands of waxed paper (15 x 2 cm) onto which females could oviposit, and provided with a source of food (5% sucrose solution). The oviposited eggs were collected daily and incubated in plastic boxes (15 x 12 x 6 cm) for ~5 days until the eggs hatched. Using a fine brush, newly hatched larvae were transferred to small plastic boxes (4 x 3 x 2 cm) containing semi-artificial diet. Larvae were checked daily until pupation, and the same procedure as described before was followed.

### Effect of gamma radiation on adult fertility and fecundity

*Lobesia botrana* pupae were removed from the small rearing boxes and placed in small transparent plastic tubes (length: 8 cm, diameter: 1 cm) and allowed to emerge at the above-mentioned rearing conditions. Newly emerged virgin adults (< 24 hr old) were sexed and exposed to gamma radiation. A Cobalt ^60^ gamma-cell (Issledova gamma Irradiator, Techsna-bexport, www.tenex.ru ) with a dose rate of 16.6 Gy/min was used to administer doses of 50, 100, 150, 200, 300, 350, and 400 Gy. The absorbed dose was measured using an alco-holic chlorobenzene dosimeter. After irradiation, each irradiated moth was placed in a transparent plastic Petri dish (9 cm diameter) with an unirradiated adult of the opposite sex (n = 45/petri dish, 1♀and 1♂for each tested dose). A control group was handled in the same way as irradiated moths but was not exposed to gamma radiation. Thus, three types of crosses were made at each dose (irradiated ♀by unirradiated ♂, unirradiated ♀by irradiated ♂, and unirradiated ♀by unirradiated ♂(control)). A 5% sucrose solution was provided as a food source to each moth pair in small cups fixed inside the Petri dish with a cotton wick soaked in the sugar solution. The moths were allowed to mate and lay eggs until the females died. *Lobesia botrana* does not require the stimulus of a host plant to initiate mating and ovipostion, thus the inner Petri dish surface served as the oviposition substrate. Females were dissected to determine their mating status by identifying the presence of spermatophores in the bursa copulatrix. Eggs deposited by each female were held separately at the previously described conditions to allow for complete egg development and larval eclosion. The fecundity (number of eggs laid per female) and fertility (number of eggs that hatched) were counted per pair at each tested dose. Each female that failed to oviposit fewer than 10 eggs throughout the entire experimental period was discarded in order to homogenize the variance. Sterility was expressed as the percentage of hatched eggs. The test consisted of three replicates of each cross at each dose of radiation, each consisting of 15 moth pairs.

### Effect of gamma radiation on developmental time, mortality, and sex ratio of F1 progeny

Newly emerged adult males (< 24 hr old) were irradiated at substerilizing doses of 100, 150, and 200 Gy (n = 45 males for each dose). At each examined dose, a group of newly emerged adult males were taken as a control. Irradiated and control males were paired individually with newly emerged females and left together until death. The oviposited eggs were collected, counted, and left to determine percentage of hatched eggs. One hundred to 150 newly hatched larvae were taken from irradiated and control groups. These larvae were fed singly on adequate artificial diet pieces (~5 g/larva). The developmental time of the F1 progeny from egg hatching to adult emergence, the number of emerged adults, and the sex ratio were determined. The tests were repeated so that there were a total of three replications with 15 pairs per replicate for each dose.

### Fertility and fecundity of F1 progeny of 150-Gy-irradiated males inbred or crossed to irradiated and unirradiated moths

Newly emerged *L* . *botrana* males were irradiated with a dose of 150 Gy and crossed to virgin unirradiated females. The deposited eggs were collected and allowed to hatch, and sterility and fecundity in the F1 generation were calculated. F1 neonates from 150-Gy-irradiated males crossed with unirradiated females were placed on artificial diet pieces. Pupae were collected and all emerging F2 adults were either inbred or paired singly with 150-Gy-irradiated or unirradiated counterparts of the opposite sex (n = 45 pairs for each cross). Moths were allowed to mate and lay eggs. Eggs were collected and incubated. The sterility and fecundity in the F2 generation were calculated. Longevity for the F1 and F2 pairs was recorded. Moreover, the percentage of sterility index was calculated using the formula of Toppozada et al. (1966) :

$\% \text{Sterlity} = [1 - (\text{F}_\text{t} \times \text{Fe}_\text{t}/\text{F}_\text{c}\times \text{Fe}_\text{c})] \times 100$

where Ft = fecundity of treated females; Fet = fertility of treated females; Fc = control fecundity; and Fec = control fertility. The experiment was conducted three times for each cross with 15 pairs per replicate.

### Statistical analysis

All statistical analyses were performed using Stat-View 4.02 version ( Abacus Concept 1994 ) at the 5% level ( *p* < 0.05). An ANOVA was carried out to evaluate the differences between the means. Significant ANOVAs were followed by the protected least significant method. Student’s *t* -test was used to evaluate the differences between two treatments. Differences in the sex ratio were determined with a chisquare test.

## Results

### Effect of gamma radiation on adult fertility and fecundity

**Figure 1.**
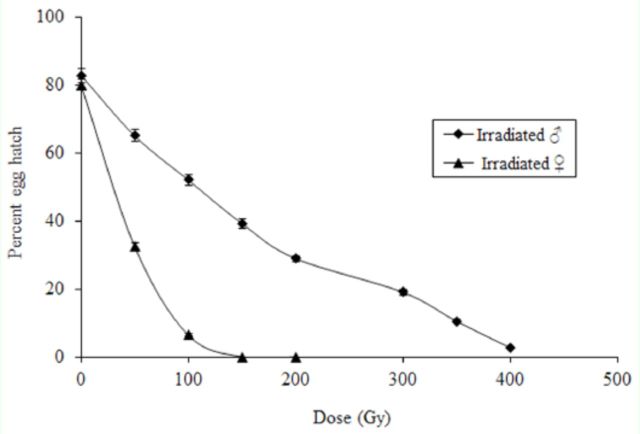
Mean percentage of hatched eggs when *Lobesia botrana* adults were treated with gamma radiation and crossed to unirradiated counterparts of the opposite sex. Bars indicate standard errors. Mean of three replicates for each dose, 15 moth pairs per replicate. High quality figures are available online.

Figure 1 presents the relationship between the applied doses of gamma radiation and the percentage of hatched eggs when *L* . *botrana* virgin males and females were irradiated and crossed to unirradiated counterparts of the opposite sex. The hatchability of the control eggs (~80%) was different from that of the irradiated moths, irrespective of the applied dose and the gender irradiated (e.g., between 0 and 100 Gy, *t* = 15.9, df = 31, *p* < 0.0001, when an irradiated male was mated with an unirradiated female). A residual fertility of 2.7% was recorded when 400-Gy-irradiated males were mated with unirradiated females.

The percentage of hatched eggs sharply declined when irradiated females were paired with unirradiated males ( *F* = 2367.1 df = 2, 102; *p* < 0.0001), indicating that the dose effect was greater for irradiated females than for irradiated males. Thus, the dose-response curve illustrates that *L* . *botrana* females became completely sterile with a dose between 100 and 150 Gy ( Figure 1 ).

**Figure 2.**
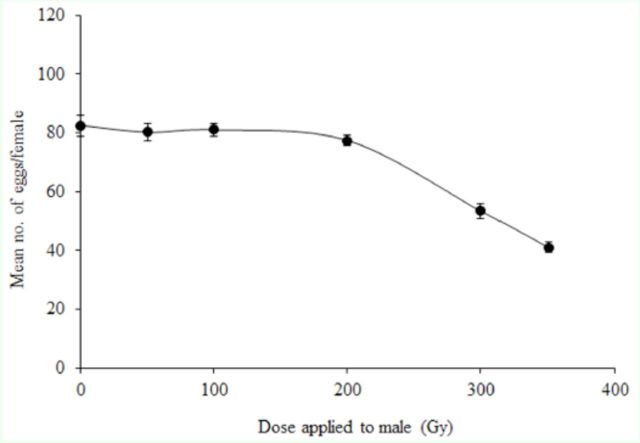
Effect of gamma radiation on fecundity (mean number of eggs per female) of *Lobesia botrana* females mated with irradiated males. Bars indicate standard errors. Mean of three replicates for each dose, 15 moth pairs per replicate High quality figures are available online.

There was no noticeable difference between the mean number of eggs laid by females that mated with unirradiated males (control) and females that mated with males irradiated at 50, 100, and 200 Gy. In contrast, at 300 and 350 Gy the mean number of eggs per female was significantly lower than that of the control ( *F* = 48.6; df = 5, 66; *p* < 0.0001) ( Figure 2 ).

### Effect of gamma radiation on developmental time, mortality, and sex ratio of F1 progeny

The mean developmental time of F1 progeny of irradiated male parents was significantly higher than that of the control (at 200 Gy, *t* = 5.8; df = 59; *p* < 0.0001). However, the developmental time of the F1 progeny of 100-Gy-irradiated male parents did not differ from that of F1 progeny of 150 and 200-Gy-irradiated sires. The mean percentage of mortality of the F1 progeny of irradiated male parents was higher than that of the control (at 100 Gy, *t* = 13.4; df = 49; *p* < 0.0001). When *L* . *botrana* males were irradiated with 100, 150 and 200 Gy, the percent mortality of their F1 progeny was similar. By irradiating the males with 100, 150, and 200 Gy, the ratio of their F1 males to F1 females was significantly higher than that of the F1 progeny of the control ( *X ^2^* = 9.1, df= 1, *p* < 0.05). However, sex ratios of F1 progeny did not differ significantly among the irradiated treatments.

### Fecundity and fertility of F1 progeny of 150-Gy-irradiated males crossed to irradiated and unirradiated moths

Fecundity and percentage of hatched eggs of *L* . *botrana* parental generation was significantly affected by the dose of radiation used (150 Gy). However, adult longevity was not affected by the applied dose ( Table 2 ). F1 females crossed to unirradiated males had residual fertility comparable to that obtained from 150-Gy-irradiated males mated with unirradiated females. A significant reduction in the percent of hatched eggs with high sterility index was recorded when F1 males were crossed to their female siblings ( *F* = 200.2; df = 4, 131; *p* < 0.0001). In F1 generation crosses, the fecundity of crosses between F1 males and unirradiateds females and F1 males and F1 females was significantly lower than that of the other crosses tested ( *F* = 65.9; df = 4, 127; *p* < 0.0001). The mean longevity of F1 adults was significantly lower than their irradiated parents (e.g., for male moths, *F* = 8.8; df = 4, 127; *p* < 0.0001).

**Table 1.**
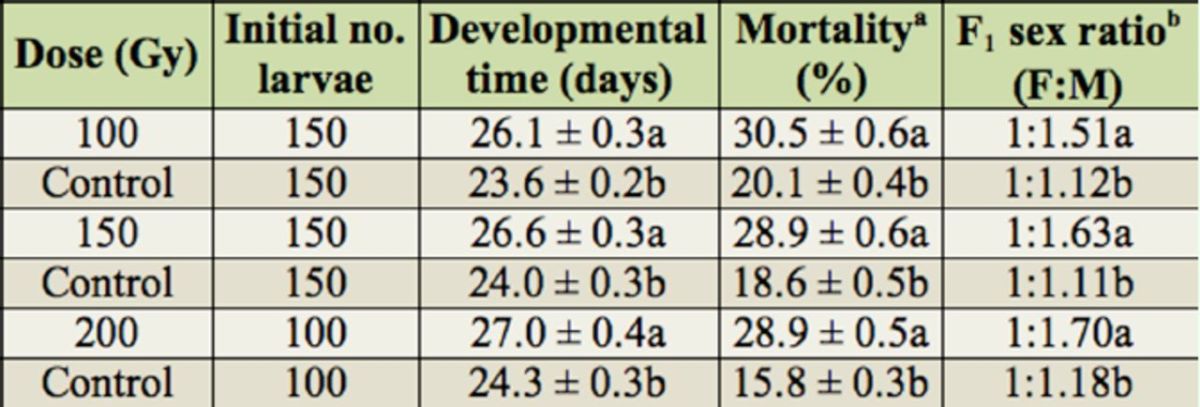
Effects of gamma radiation on developmental time, mortality (mean ± SE), and sex ratio of the F1 progeny of the male parents of *Lobesia botrana* when they were irradiated at different doses. Larvae were reared at 25º C with a photoperiod of 16:8 L:D.

Means within a column for each applied dose followed by the same letter are not significantly different at *p* < 0.05 (Student *t* -test). ª Number of unemerged adults per initial number of tested larvae. b Ratios within a column for each applied dose followed by the same letter are not significantly different at *p* < 0.05 (Chisquare test). F, female; M, male. Mean of three replicates for each dose, 15 moth pairs per replicate.

**Table 2.**
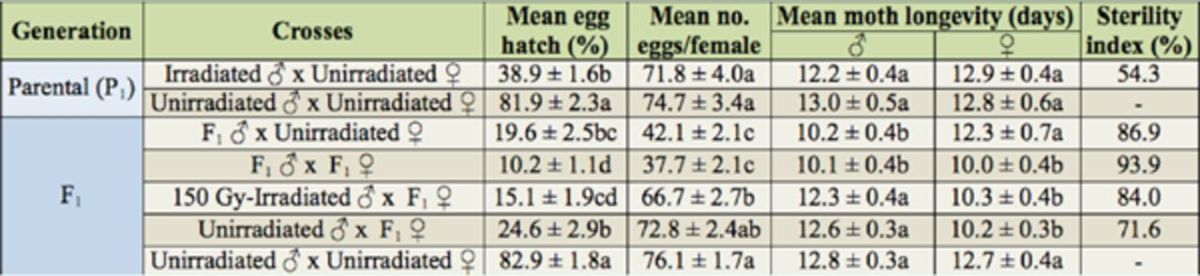
Means (± SE) of hatched eggs, number of eggs/female, moth longevity, and sterility index when *Lobesia botrana* males were irradiated with 150 Gy and crossed to fertile counterparts (parental generation (P1)), and when F1 adults resulting from irradiated male crossed to unirradiated female were inbred or crossed to 150-Gy-irradiated and unirradiated moths.

Means within a column for each generation followed by the same letter are not significantly different at *p* < 0.05 (Fisher PLSD). % sterility index = [1 -(Ft x Fet / Fc x Fec)] x 100. Ft = fecundity of irradiated females; Fet = fertility of irradiated females; Fc = control fecundity; and Fec = control fertility. Mean of three replicates for each cross, 15 moth pairs per replicate.

## Discussion

*Lobesia botrana* adults responded to increasing doses of gamma radiation with a decline in female fecundity and male fertility. Furthermore, it was found that females were more radiosensitive than were males, reduction in female fecundity was greater when they were crossed with males treated at high doses, and F1 progeny from irradiated males were less fertile than their irradiated parents. These findings are in agreement with those reported for other Lepidoptera species ( Saour and Makee 1997 ; Carpenter et al. 2001 ; Makee and Saour 2004 ). However, comparing the results of *L* . *botrana* male fertility with those obtained from *C. pomonella* ( Bloem et al. 2007 ) and the *T. leucotreta* ( Carpenter et al. 2007 ), we found that *L* . *botrana* was more ra-dioresistant than these two tortricid species (400 vs 350 Gy to obtain full sterility). However, *L* . *botrana* was less resistant to radiation than the potato tuber moth, *Phthorimaea operculella* ( Saour and Makee 1997 ), and *C. cactorum* (400 vs 500 Gy) ( Carpenter et al. 2001 ).

Successful application of SIT/F1 in Lepidoptera is achieved by selecting a treatment dose of radiation that fully sterilizes females, to avoid increasing host plant damage, while only partially sterilizing males to maintain mating competitiveness and produce F1 progeny highly sterile when mated with feral females ( Tate et al. 2007 ). According to the results of our study, the dose of radiation providing these attributes in *L* . *botrana* was found to be 150 Gy. At this radiation dose, the fertility of irradiated males mated with unirradiated females was reduced by ~61%, while only an average of 20% of eggs hatched for F1 males that mated with unirradiated females. Moreover, there was no impact of radiation on the parental generation female fecundity, and females that mated with irradiated males laid nearly as many eggs as did the controls.

The effects of radiation and inherited sterility on the reproduction of *L* . *botrana* were similar to those described for other species of Lepidoptera, i.e., reduced survival of larvae, the delay in the developmental time from F1 neonate to adult, and the shift of sex ratio in favor of males in the F1 generation. However, the sex ratio distortion of the F1 progeny in favor of males was one of the benefits of inherited sterility.

There was a skewed sex ratio in favor of males for F1 *L* . *botrana* adults. These results are in agreement with those reported previously for *P* . *operculella* ( Makee and Saour 1997 ), *Spodoptera litura* ( Ramesh et al. 2002 ), and *C* . *leucotreta* ( Hofmeyr et al. 2005 ). On the other hand, Carpenter et al. (2001) did not detect a skewed sex ratio in favor of male offspring for the *C* . *cactorum* F1 adults. It is worth noting that the fecundity of F1 females obtained in our study from male parents irradiated with 150 Gy and mated with unirradiated males was not significantly different from that of unirradiated moths. This finding is similar to those reported by Makee and Saour (1997) and Bloem et al. (2003) in F1 progeny of *P* . *operculella* and *C* . *leucotreta* , respectively.

In all current SIT/F1 sterility programs against Lepidoptera, both males and females are mass-reared, irradiated, and then released into the targeted area because no practical method is available to separate the adult moths by gender ( Bloem et al. 2007 ; Blomefield et al. 2011 ). Moreover, the irradiated moths are released continuously from the beginning of the season, thus the possibility of crosses involving 150-Gy-irradiated males with F1 females and F1 males with their female counterparts could occur. The results of our study showed that the fertility of unirradiated males crossed to F1 females did not differ significantly from that of the cross between F1 males mated with unirradiated females, which suggests that *L* . *botrana* F1 females inherited the deleterious effects from their irradiated male parents. High values of unhatched eggs and sterility index were obtained when F1 males were mated with either F1 or unirradiated females and when 150-Gy-irradiated males were mated with F1 females.

The results presented here provide a starting point for developing SIT/F1 sterility program against *L* . *botrana* .

## Abbreviations

SIT: sterile insect technique

## References

[R1] Abacus Concepts . 1994 . StatView , Version 4.02. Abacus Concepts .

[R2] Akyol B Aslan MM . 2010 . Investigations on efficiency of mating disruption technique against the European grapevine moth ( *Lobesia botrana* Den. &. Schiff.) (Lepidoptera: Tortricidae) in vineyard, Turkey . Journal of Animal and Veterinary Advances 9 : 730 – 735 .

[R3] Bakri A Mehta K Lance DR . 2005 . Sterilizing insects with ionizing radiation. In: Dyck VA, Hendrichs J, Robinson AS, Editors . Sterile Insect Technique: Principles and Practice in Area-wide Integrated Pest management . pp. 233 – 268 . Springer.

[R4] Bloem S Carpenter JE Hofmeyr JH . 2003 . Radiation biology and inherited sterility in false codling moth (Lepidoptera: Tortricidae) . Journal of Economic Entomology 96 : 1724 – 1731 . 14977109 10.1603/0022-0493-96.6.1724

[R5] Bloem S McCluskey A Fugger R Arthur S Wood S Carpenter JE . 2007 . Suppression of the codling moth *Cydia pomonella* in British Columbia, Canada using an area-wide integrated approach with an SIT component. In: Verysen MJB, Robionson AS, Hendrichs J, Editors . Area-Wide Control of Insect Pests . pp. 591 – 601 . Springer .

[R6] Blomefield T Carpenter JE Verysen MJB . 2011 . Quality of mass-reared codling moth (Lepidoptera: Tortricidae) after long-distance transportation: 1. logistics of shipping procedures and quality parameters as measured in the laboratory . Journal of Economic Entomology 104 : 814 – 822 . 21735898 10.1603/ec10238

[R7] Carpenter JE Bloem S Bloem KA . 2001 . Inherited sterility in *Cactoblastis cactorum* (Lepidoptera: Pyralidae) . Florida Entomologist 84 : 537 – 542 .

[R8] Carpenter JE Bloem S Marec F . 2005 . Inherited Sterility in Insects. In: Dyck VA, Hendrichs J, Robinson AS, Editors . Sterile Insect Technique: Principles and Practice in Area-wide Integrated Pest management . pp. 115 – 146 . Springer .

[R9] Carpenter JE Bloem S Hofmeyr H . 2007 . Area-wide control tactics for the false codling moth *Thaumatotibia leucotreta* in South Africa: a potential invasive species. In: Verysen MJB, Robionson AS, Hendrichs J, Editors . Area-Wide Control of Insect Pests . pp. 351 – 359 . Springer.

[R10] Hight SD Carpenter JE Bloem S Bloem KA . 2005 . Developing a sterile insect release program for *Cactoblastis cactorum* (Berg) (Lepidoptera: Pyralidae): effective overflooding ratios and release-recapture field studies . Environmental Entomology 34 : 850 – 856 .

[R11] Hofmeyr JH Carpenter JE Bloem S . 2005 . Developing the sterile insect technique for *Cryptophlebia leucotreta* (Lepidoptera: Tortricidae): influence of radiation dose and release ratio on fruit damage and population growth in the field cages . Journal of Economic Entomology 98 : 1924 – 1929 . 16539115 10.1603/0022-0493-98.6.1924

[R12] Ifoulis AA Savopoulou-Soultani M. 2004 . Biological control of *Lobesia botrana* (Lepidoptera: Tortricidae) larvae by using different formulations of *Bacillus thuringiensis* in 11 vine cultivars under field conditions . Journal of Economic Entomology 97 : 340 – 343 . 15154453 10.1093/jee/97.2.340

[R13] Ioriatti C Anfora G Tasin M De Cristofaro A Witzgall P Lucchi A. 2011 . Chemical ecology and management of *Lobesia botrana* (Lepidoptera: Tortricidae) . Journal of Economic Entomology 104 : 1125 – 1137 . 21882674 10.1603/ec10443

[R14] Irigaray FJ Marco V Zalom FG Pérez-Moreno I. 2005 . Effects of methoxyfenozide on *Lobesia botrana* Den & Schiff (Lepidoptera: Tortricidae) egg, larval and adult stages . Pest Management Science 61 : 1133 – 1137 . 15962346 10.1002/ps.1082

[R15] Irigaray FJ Moreno-Grijalba F Marco V Pérez-Moreno I. 2010 . Acute and reproductive effects of Align ^®^ , an insecticide containing azadirachtin, on the grape berry moth, *Lobesia botrana* . Journal of Insect Science 10 : 33 . Available online: www.insectscience.org/10.33 20578954 10.1673/031.010.3301 PMC3014762

[R16] Jang EB McInnis DO Kurashima R Woods B Suckling DM . 2012 . Irradiation of adult *Epiphyas postvittana* (Lepidoptera: Tortricidae): egg sterility in parental and F1 generations . Journal of Economic Entomology 105 : 54 – 61 . 22420255 10.1603/ec11135

[R17] LaChance L.E. Graham CK . 1984 . Insect radiosensitivity: Dose curves and dose-fractionation studies of dominant lethal mutations in the mature sperm of 4 insect species . Mutation Research 127 : 49 – 59 . 6539419 10.1016/0027-5107(84)90139-8

[R18] Makee H Saour G . 1997 . Inherited effects in F1 progeny of partially sterile male *Phthorimaea operculella* (Lepidoptera: Gelechiidae) . Journal of Economic Entomology 90 : 1097 – 1101 .

[R19] Makee H Saour G . 2004 . Efficiency of inherited sterility technique against *Phthorimaea operculella* Zeller (Lepidoptera: Gelechiidae) as affected by irradiation of females . Journal of Vegetable Crop Production 10 : 11 – 22 .

[R20] Murakami A Imai HT . 1974 . Cytological evidence for holocentric chromosomes of the silkworms, *Bombyx mori* and *B* . *mandarina* (Bombycidae, Lepidoptera) . Chromosoma 47 : 167 – 178 . 4141955 10.1007/BF00331804

[R21] Ramesh K Garg AK Seth RK . 2002 . Interaction of substerilizing gamma radiation and Thiodicarb treatment for management of the tobacco caterpillar *Spodoptera litura* . Phytoparasitica 30 : 7 – 17 .

[R22] Saour G Makee H . 1997 . Radiation induced sterility in male potato tuber moth *Phthorimaea operculella* Zeller (Lep., Gelechiidae) . Journal of Applied Entomology 121 : 411 – 415 .

[R23] Soopaya R Stringer LD Woods B Stephens AEA Butler RC Lacey I Kaur A Suckling DM . 2011 . Radiation biology and inherited sterility of light brown apple moth (Lepidoptera: Tortricidae): developing a sterile insect release program . Journal of Economic Entomology 104 : 1999 – 2008 . 22299363 10.1603/ec11049

[R24] Tate CD Carpenter JE Bloem S . 2007 . Influence of radiation dose on the level of F1 sterility in the cactus moth, *Cactoblastis cactorum* (Lepidoptera: Pyralidae) . Florida Entomologist 90 : 537 – 544 .

[R25] Thiery D Moreau J . 2005 . Relative performance of European grapevine moth ( *Lobesia botrana* ) on grapes and other hosts . Oecologia 143 : 548 – 557 . 15791428 10.1007/s00442-005-0022-7

[R26] Toppozada A Abdallah S Eldefrawi ME . 1966 . Chemosterilization of larvae and adults of the Egyptian cotton leafworm *Prodenia litura* , by apholate, metepa, and tepa . Journal of Economic Entomology 59 : 1125 – 1128 .

[R27] Tothova A Marec F . 2001 . Chromosomal principle of irradiation-induced F1 sterility in *Ephestia kuehniella* (Lepidoptera: Pyralidae) . Genome 44 : 172 – 184 . 11341727 10.1139/g00-107

[R28] Varela LG Smith RJ Cooper ML Hoenisch RW . 2010 . European Grapevine Moth, *Lobesia botrana* , in Napa Valley vineyards . Practical Winery and Vineyard (March/April): 1 – 5 .

[R29] Yadav SK . 2010 . Pesticide applications-threat to ecosystems . Journal of Human Ecology 32 : 37 – 45 .

